# The Atlanta Medical Society

**Published:** 1882-07

**Authors:** 


### THE ATLANTA MEDICAL SOCIETY.

*Reported for the Atlanta Medical Register.*

ATLANTA, May 27, 1882.

The President, Dr. W. S. Armstrong, presiding.

Dr. Jno. Thad. Johnson reported a case of ulceration of the rectum, possibly involving the lower bowel. The commencement of the attack was an acute dysentery, and it may now with propriety, perhaps, be called chronic dysentery. The disorder dates back six or eight months. Has been under his treatment four months. The symptoms were slight at first—consisting of light colicky pains and a trivial diarrhœa, subsequently, as stated, they became dysenteric. The patient is a stout, strong man of careless habits and reckless disposition, impatient of restraint and accustomed to obey the dictates of inclination in the gratification of his appetite and desires of all kinds. After any imprudence—as excess in exercise or at table the discharges from the anus will consist of some fecal matter, then blood, mucus and pus. He experiences severe neuralgic pains over the abdomen, thighs, penis and testicles. He suffers in this manner extremely. Made a thorough rectal examination by aid of a speculum and reflected light. Found several ulcers. Medication, *per orem*, including strong astringents, was productive of no appreciable effect. Is compelled to use some opium now. Daily quantity about three-quarters of a grain of morphine. Has made the attempt to restrict his diet to milk, but without success. He is at present using injections of nitrate of silver, strength twenty grains to the ounce of water, of which solution one and a half ounces are injected into the rectum and retained for twenty-four hours. This results in the dejection of molded fœces and the good effect continues for several days. Then the same proceeding with the same result is repeated. Has tried a sixty-grain to ounce solution, but it gives intense pain. Now uses the twenty-grain solution every other day and the man remains reasonably comfortable. If it should be omitted for four or five days all the symptoms return and the benefits of the former applications are lost.

Dr. G. G. Roy indorsed the plan of treatment adopted. Thinks internal treatment *per orem* worthless. The nitrate of silver is the best and most reliable agent. It would be well, he thought, to inject warm water containing some tar soap, after each action of the bowels.

Dr. F. H. O'Brien inquired if there was not danger of toxic effects from absorption of the caustic?

Dr. Johnson, thought the diseased state of the mucous coat would impair even the ordinary power of absorption, which, at best, he considers very slight in the lower bowel.

Dr. W. S. Armstrong advocated milk diet exclusively; rest, complete—and urged the importance of seeing that the work of the stomach is well performed.

Dr. James B. Baird suggested forcible dilatation of the sphincter ani and the application of a stronger solution or of the solid stick of nitrate of silver to the ulcers themselves. This method of treatment would, of course, be only available for ulcerated surfaces below the internal sphincter.

Dr. F. H. O'Brien approved of a stronger solution than twenty grains to the ounce in this case, and spoke favorably of applying the vapor of chloroform after every injection, for the purpose of relieving pain. It may be done by placing a sponge saturated with chloroform in an ordinary syringe, and by compressing it with the piston the vapor will be driven through the nozzle into the bowel. It is a valuable resource for the relief of tenesmus in dysentery.

Dr. O'Brien, continuing, reported a case of pernicious anæmia or leucocythemia, as follows: The patient came to this city five or six weeks ago. Had made a slow recovery after confinement. Was very feeble and presented a waxy appearance. Two weeks after her arrival, one night, she discharged from her bowels half a gallon of dark colored, offensive clots resembling in size, shape and color stewed prunes. The patient died a few hours after the hemorrhage.

He also reported the case of a child, four years of age, suffering from chronic nephritis. One year ago had measles; soon after recovery was vaccinated, and a very sore arm, for several weeks, resulted. Since then has had urticaria. The child is most of the time in a dull stupor, passes an excessive quantity of urine. Specific gravity—decanted—1001. Contains casts, blood, mucus and some pus, besides granular matter and a trace of albumen. This disease is not a common sequel of measles.

Dr. G. G. Roy, referring to a case reported several weeks ago as a false conception, said that the lady had recently expelled the contents of the womb, accompanied by some pain and preceded by slight hemorrhage for several hours. The expelled mass was about six inches in its longest diameter, and when laid open revealed the abdominal and thoracic viscera of a fœtus, but no head or features were discernable. Slight hemorrhage continued for several days, and the patient is now doing well.

Dr. Roy also reported the cases of two children—girls—aged four and six years respectively. They both had a vaginal discharge resembling gonorrhœa. Has not made an inspection of the parts. The older of the sisters had the same trouble two years ago. Mild astringent washes then relieved the symptom. The same plan of treatment has not proved so successful in the present instance. Thought the discharge might be due to worms, and prescribed calomel, santonin and spigelia with the view of expelling them. The older child discharged two, but the younger none. The annoying disorder continues.

Dr. F. H. O'Brien stated that rectal worms frequently caused this trouble by getting into the vagina.

Dr. Baird confirmed this view, and expressed the opinion that the symptom in the cases narrated was referable, in all probability, to the migration of the small seat or maw worm—ascaris vermicularis—from the rectum into the folds of the vaginal mucous membrane and of the pudenda. Close inspection will probably discover the slender, maggot-like intruders. Has frequently encountered similar cases, and always obtained relief by the internal administration, sometimes in repeated courses, of santonin and calomel, supplemented by salt water bathing. These worms are tenacious and are rapidly reproduced. Patience and careful attention, with good hygienic management are often necessary to effect a permanent cure.

Dr. W. S. Armstrong reported the following case:

Six weeks ago Mrs. S., aged twenty-three, gave birth to her first child. Her attending physician informed me that the labor was rapid and the child large. Rupture of the perineum, extending through the sphincter ani and involving a small portion of the recto vaginal septum ensued.

She has been unable to control the movements from the bowels, and as she expressed it, the evacuation would "pass both ways."

I was invited to examine the case, ten days ago, with the view of restoring the ruptured parts by operation. While the extent and depth of the rupture rendered it a formidable operation, it was decided to perform it on Thursday last, 23d inst.

The patient was put under ether and placed in position. The cicatrized surface covering the lacerated parts was rapidly removed by a pair of scissors in the following manner: A small opening was made at the point of union of skin and cicatricial tissue. Into this opening the scissors were inserted and the dissection gradually accomplished by opening and closing the blades. The escape of blood at the point of entrance was only a few fluid drachms, and that beneath the membrane, by giving it a dark color, formed a guide to the point of the scissors. The dissection was continued from the lacerated perineum to the vaginal mucous membrane covering the lower three-fourths of an inch of the recto-vaginal septum—this latter step being necessary to secure union between the perineum and recto-vaginal septum, and thus prevent a fistulous opening between the vagina and the rectum. I was careful, also, to freshen up the extremities of the ruptured sphincter.

This being accomplished, three silver wire sutures were passed deeply into the perineum on one side, through the freshened surface of the recto-vaginal septum, and out at the opposite side of the perineum. They were then drawn sufficiently tight to adapt the edges without compressing the freshened surfaces. Two other superficial sutures were placed above these to approximate the upper part of the perineum.

Thus far the case is progressing favorably.

